# Supplementary material for: Broad susceptibility of Candida auris strains to 8-hydroxyquinolines and mechanisms of resistance
Source: mBio. 2023 Jul 26;14(4):e01376-23. doi: 10.1128/mbio.01376-23 (PMC10470496; doi:10.1128/mbio.01376-23)
Supplement: File S7 — Supplemental text explaining the Minority Report and alternative R-based mutation identification outputs. [file mbio.01376-23-s0007.docx]

**Lohse et al. File S7: Supplemental text explaining the Minority Report and alternative R-based mutation identification outputs.**

**Column Explanations for “Full R Call Set” and “R Call Q>200” tabs**

The following information may be helpful when examining the data in the “Full R Call Set” or “R Call Q>200” tabs in File S6.

Column Explanations

Order (Column A) – A column for returning data organization to the initial order, numbers in this column are arbitrary.

CHROM (Column B) – Chromosome (or Contig) name

POS (Column C) – Position of variant allele

REF (Column D) – Reference allele

ALT (Column E) – Alternate allele

QUAL (Column F) – Quality (Phred-scaled quality score for the assertion made in ALT)

Q200 (Column G) – Is the Quality Score (Column F) for the assertion at least 200? (Yes or No)

Read50 (Column H) – Is the Reads_2 Number (Column P) at least 50 for the assertion? (Yes or No)

INDEL (Column I)– Is the variant an indel (True equals yes, False equals no)

SIZE (Column J) – Length of the alternate allele subtracted by the length of reference allele

READS (Column K) – Value taken from the ‘DP’ field of VCF file

REF.F, REF.R, ALT.F, ALT.R (Columns L-O) – Values taken from the ‘DP4’ field of VCF file

READS_2, REF_2, ALT_2 (Columns P-R) – Sums based on numbers from ‘DP4’ field of VCF file

GENE1, GENE2, NOTE, STRAND1, STRAND2, DISTANCE1, DISTANCE2 (Columns S-Y). GENE1 and GENE2 are systematic name(s) of the gene or genes. NOTE indicates whether the variant is located in a gene, an intergenic region, or between a gene and the end of a chromosome. STRAND1 and STRAND2 indicate the strand that GENE1 and GENE2 are located on. DISTANCE1 and DISTANCE2 are the distance from the start codon of gene or the nearest coordinate of the gene depending on the location of the variant. There are four possibilities for the information present in these columns depending on the nature of the variant.

1. The variant is located in a gene. The systematic name of the Gene in GENE1, GENE2=NA, NOTE=Gene, STRAND1 is the strand of GENE1, STRAND2=NA, DISTANCE1 is the distance from the start codon of the gene (considers strand), DISTANCE2=NA
2. The variant is located in an intergenic region – GENE1 is upstream of the variant, GENE2 is downstream of the variant, NOTE=Intergenic, STRAND1 is the strand of GENE1, STRAND2 is the strand of GENE2, DISTANCE1 is the distance from last coordinate of GENE1 (does not consider strand), DISTANCE2 is the distance from first coordinate of GENE2 (does not consider strand)
3. The variant is located before the first gene on a chromosome (or contig), GENE1=NA, GENE2 is downstream of variant, NOTE= Intergenic_CHRstart, STRAND1=NA, STRAND2 is strand of GENE2, DISTANCE1=NA, DISTANCE2 is the distance from first coordinate of GENE2 (does not consider strand)
4. The variant is located after the last gene on a chromosome (or contig), GENE1 is upstream of variant, GENE2=NA, NOTE= Intergenic_CHRend, STRAND1 is strand of GENE1, STRAND2=NA, DISTANCE1 is the distance from last coordinate of GENE1 (does not consider strand), DISTANCE2=NA

NAME1/NAME2 (Columns Z-AA) – Common names if they exist for GENE1/GENE2, organized in a format of *C.auris*_*C.albicans*_*S.cerevisiae*. We note the homology calls across species were largely assigned in an automated manner based on the Pillars file from the *Candida* Gene Order Browser (CGOB). Likewise, the homology calls between the *C. auris* B8441 and B11221 genomes were based on a largely automated process based on protein sequence identity between the B8441 and B11221 ORF Translation files from the *Candida* Genome Database (CGD). Given the nature of these assignments, we recommend manually confirming homology for any genes of interest.

Samples (Column AB) – The sample name.

Genome (Column AC) – Reference genome used for this sample based on the parent strain.

Culture (Column AD) – Culture Name-Passage Number information for the sample. We have reformatted A8, B8 and H8 to A08, B08, and H08 respectively to allow for a more intuitive result when sorting by this column.

Population (Column AE) – Whether the culture that the sample was harvested from was started from a population of cells (True) or a single colony (False)

Note (Column AF) – The type of sample or treatment type. “Parent” refers to the parent strains used for the experimental evolution study and are a way to identify places where the starting strains differed from the reference genomes used. “DMSO” refers to the DMSO treated control cultures. “CQ” refers to the Clioquinol treated cultures.

PAR_VAR (Column AG) – Is this variant found (True) or not found (False) in the parent strain. Filtering this column so that only “FALSE” is displayed will remove most (see note for next item for exceptions) of the variants that were present in the starting strain (relative to the reference genome) and as such did not arise during this experiment.

DMSO_VAR (Column AH) – Is the variant found (True) or not found (False) in the DMSO treated control samples. Theoretically, filtering this column so that only “FALSE” is displayed will remove variants that also arose in cells not treated with Clioquinol which would represent variants related to the general experimental setup rather than exposure to Clioquinol. In practice, we note that the analysis pipeline used to generate this file will sometime assign variants in a highly repetitive region to different locations within that region and, as such, some variants (relative to the reference genome) that were present in the starting strain will not be removed by the “PAR_VAR” filtering step. As such, we recommend using this column in addition to “PAR_VAR” to help filter out said variants.

**Column Explanations for the “SNP_#_S#” tabs**

The following information may be helpful when examining the Minority Report generated output data in the 22 tabs with a “SNP_#_S#” format name in File S6. We note that Minority Report only identifies variants within gene regions so these reports will not contain the intergenic or end of chromosome variants reported in the “Full R Call Set” tab. As will be noted in more detail below where relevant, due to the way in which the Minority Program works, there can be changes in the way data is reported for a variant when genes are located on the reverse strand and/or when an indel is the cause of sequence variation. For these files, the Experimental Sample is the Clioquinol or DMSO treated sample and the Parent or Parental Strain or Sample are the samples from the starting strain that was used as the basis for comparison within Minority Report.

Chromosome (Column B) – Chromosome or contig the variant is located on.

genomic_mutation (Column C) – Position and nature of the variant on the chromosome/contig indicated in column B.

gene_mutation (Column D) – Position and nature of the variant within a gene. If the gene is located on the reverse strand, the specific nature of the variation will reflect the reverse complement of that indicated in Column C (e.g. C to A in Column C but G to T in column D).

protein_mutation (Column E) – Amino acid location and nature of the mutation due to the variant. We note that when Minority Report encounters indels (see, for example, the variants around T387 for CJI97_005427 in tab SNP_14_S14), it will report a string of variants working outward from the location of the indel for each base pair/codon pairing that causes a nonsynonymous mutation with a frequency that decreases as the distance increases until the frequency reaches the threshold settings (0.9 for single cell samples, 0.1 for population samples). Furthermore, when genes are located on the reverse strand, we note that this effect will run from the indel location towards the start of the gene. As such, Minority Report sometimes has issues identifying the exact location of the premature stop codon resulting from an indel as well as the specific nature of the indel. In these cases, we manually examined the alignments and looked at the location in the Alternative R Based Mutation Identification Outputs in order to determine the nature of the variant and its effect.

mutation_counts (Column F) – Number of reads with the indicated variant in the experimental sample. We note that is value had to be above 10 for population samples.

mutation_proportion (Column G) – Proportion of the experimental reads for a given position that contained the indicated variant. This is equal to the value in Column F divided by the value in Column H. We note that this value had to be above 0.9 for single cell samples and 0.1 for population samples.

mutant_position_counts (Column H) – Total number of experimental sample reads containing the position where the variant is located, with or without the variant.

parent_mutation_counts (Column J) – Number of parental strain sample reads with the indicated variant. We note that this value had to be below 50 for population samples.

parent_mutation_proportion (Column K) – Proportion of the parental strain sample reads for a given position that contained the indicated variant. This is equal to the value in Column J divided by the value in Column L. We note that this value had to be below 0.04 for population samples

parent_position_counts (Column L) – Total number of parental strain sample reads containing the position where the variant is located, with or without the variant.

gene_id (Column N) – Systematic name of the gene.

strand (Column O) – Strand the gene is located on.

protein_length (Column P) – Length, in amino acids, of the protein encoded by the wild type gene.

Variant location alternative base information (Five groups, located in columns S-Y, AA-AG, AI-AO, AQ-AW, and AY-BE). These columns give details on all possible base pair substitutions at a given location (to A, C, G, T, or a nonACGT variant, one of these will represent the wild type sequence). We note that, although Minority Report groups these as A-gene, C-gene, G-gene, T-gene, and nonACGT-gene variants, in some case the specific base pair variants will not be in the expected columns due to the strand the gene is located on (e.g. instances of the base T at a location will appear in the “A-gene_variant” set of columns while instances of the base A will appear in the “T-gene_variant” set of columns). Using the “A-gene_variant” set of columns (columns S-Y) as location examples, these columns are…

A-gene_variant (Column S) – Position of this specific base and the nature the change, if any, resulting from having the indicated base at this position.

protein_variant (Column T) – Amino acid location and nature of the mutation (or “-“ if there would not be a mutation) due to this specific base at this position.

variant_counts (Column U) – Number of reads with this specific base in the experimental sample.

variant_proportion (Column V) – Proportion of the experimental reads for a given position that contained this specific base.

parent_variant_counts (Column X) – Number of parental strain sample reads with this specific base.

parent_variant_proportion (Column Y) – Proportion of the parental strain sample reads for a given position that contained this specific base.
