# Supplementary material for: Broad susceptibility of Candida auris strains to 8-hydroxyquinolines and mechanisms of resistance
Source: mBio. 2023 Jul 26;14(4):e01376-23. doi: 10.1128/mbio.01376-23 (PMC10470496; doi:10.1128/mbio.01376-23)
Supplement: Figure S1 — Clioquinol is fungistatic to C. auris. [file mbio.01376-23-s0008.pdf]

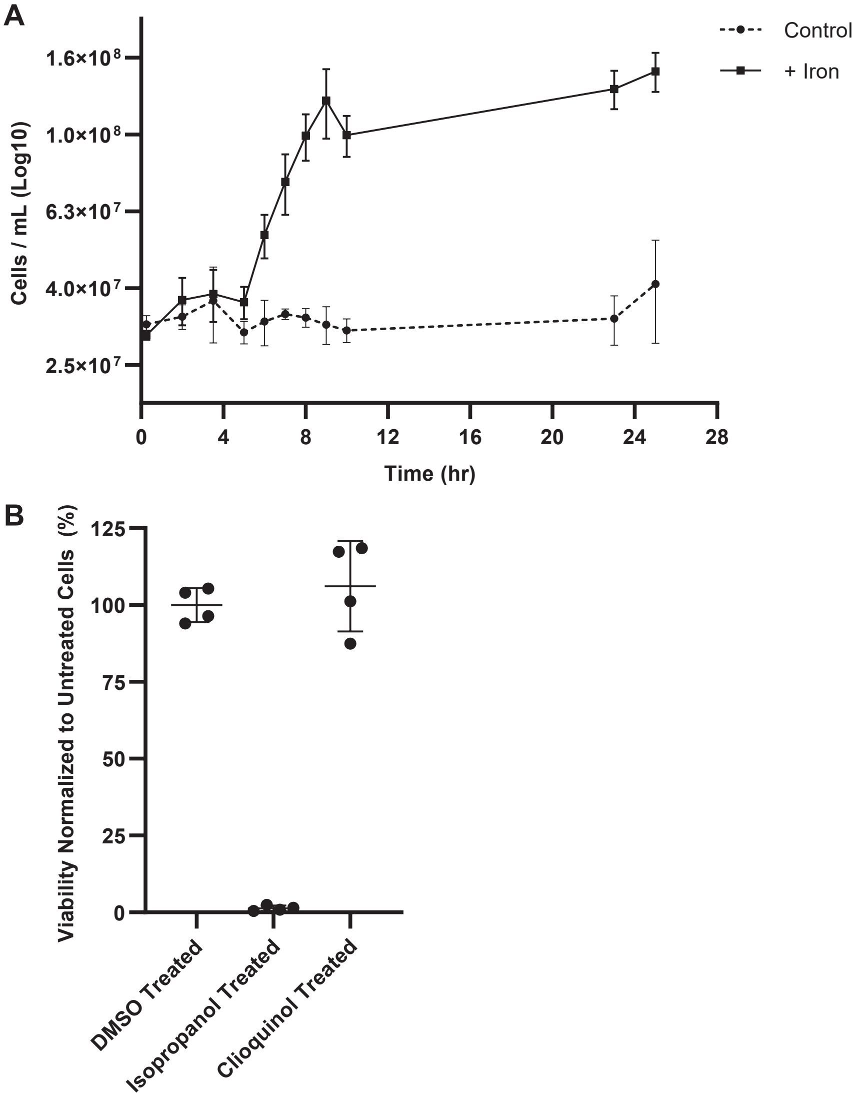

Figure S1: Clioquinol is fungistatic to *C. auris*. (A) Addition of iron restores growth of Clioquinol treated *C. auris* cells. Following a 21-hour incubation in the presence of 4 μM Clioquinol, either iron (2 μM each of iron (II) sulfate and iron (III) chloride, "+ Iron", squares and solid line) or an equivalent volume of water ("Control", circles and dashed line) was added to the cells at the 0-hour timepoint and cells were then incubated on a roller drum at 30°C for 25 hours. Cell counts were determined by flow cytometry at the indicated time points; data represent the average of four independent cultures from the same day, error bars represent the standard deviation. (B) Viability of *C. auris* after 22 hours exposure to clioquinol. Aliquots from 22-hour clioquinol (5 μM) and DMSO or 1 hour isopropanol (70%) treated cultures were washed, plated on YEPD plates, and incubated for two days at 30°C. Input cell density as determined by flow cytometry (cells/μL) and the number of colonies were both normalized to the average of their respective DMSO treated samples; normalized viability relative to the DMSO treated samples was determined by dividing the normalized CFU for each sample by the normalized input cell density. Dots represent individual replicates; the mean and standard deviation are indicated for each.
